# Understanding the Pathophysiology of Exosomes in Schistosomiasis: A New Direction for Disease Control and Prevention

**DOI:** 10.3389/fimmu.2021.634138

**Published:** 2021-06-16

**Authors:** Yue Yuan, Jianping Zhao, Min Chen, Huifang Liang, Xin Long, Bixiang Zhang, Xiaoping Chen, Qian Chen

**Affiliations:** ^1^ Division of Gastroenterology, Department of Internal Medicine at Tongji Hospital, Tongji Medical College, Huazhong University of Science and Technology (HUST), Wuhan, China; ^2^ Hepatic Surgery Center, Tongji Hospital, Tongji Medical College, Huazhong University of Science and Technology (HUST), Wuhan, China

**Keywords:** schistosomiasis, exosome, liver fibrosis, diagnosis, macrophage polarization, treatment, Th immune response

## Abstract

Schistosomiasis is a parasitic disease endemic to freshwater areas of Southeast Asia, Africa, and South America that is capable of causing serious damage to the internal organs. Recent studies have linked exosomes to the progression of schistosomiasis. These structures are important mediators for intercellular communication, assist cells to exchange proteins, lipids, and genetic material and have been shown to play critical roles during host–parasite interactions. This review aims to discuss the pathophysiology of exosomes in schistosomiasis and their roles in regulating the host immune response. Understanding how exosomes are involved in the pathogenesis of schistosomiasis may provide new perspectives in diagnosing and treating this neglected disease.

## Introduction

Schistosomiasis is caused by trematodes of the genus *Schistosoma* and is the second leading parasitic disease after malaria in terms of public health impact (1, 2). Currently, there are more than 230 million people worldwide living with this parasite (1). *Schistosoma* infection is characterized by the development of granulomas surrounding helminth eggs. In particular, *S. mansoni* and *S. japonicum* living in the mesenteric veins lay eggs; these eggs eventually reach the portal vein, resulting in portal fibrosis. For this reason, infected individuals present with portal hypertension signs and symptoms and ultimately liver failure and death (1, 3). The host immune response to schistosomiasis dramatically evolves with parasite migration and maturation in a time-dependent manner. The acute phase of infection is associated with a Th1 response that is rapidly superseded by a Th2 response reacting to a large number of eggs deposited in the liver, triggering liver tissue injury and stimulating fibroblasts to make collagen, which leads to fibrosis (1, 4–6). Current diagnostic techniques include the Kato-Katz (KK) test and immunodiagnostic methods (7), but the rate of early diagnosis remains poor; improving diagnostic tests could therefore reduce the prevalence rate of advanced schistosomiasis (8, 9).

Exosomes are nanoscale membrane-derived vesicles that transport bioactive substances, including DNA, RNA, and proteins, which can trigger downstream signaling pathways and influence both neighboring and distant cells (10–12). Recent studies have linked exosomes to the immune response and suggested that they can recruit immune cells such as macrophages, dendritic cells (DCs), and T cells to initiate the pathophysiologic processes (13, 14). Furthermore, various processes or stimulation can stimulate the release of exosomes to adapt to the circumstances or modulate the microenvironment (15). In fibrotic diseases, conditions including autophagy inhibition, lysosome dysfunction, and cytokine release can alter exosome secretion to modulate phenotypes such as hepatic stellate cell activation (16, 17). Therefore, as a disease closely related to inflammation and fibrosis, schistosomiasis may have a close relationship with exosomes, but the specific roles exosomes play in immune responses and liver fibrosis have not been elucidated. One current trend in parasitology is to study the contents and functions of exosomes to target host-parasite interactions and controlling schistosomiasis-associated immune evasion (18). This review elaborates the functional niches in which exosomes operate in schistosomiasis and the emerging prospects of targeting these vesicles in therapeutic strategies.

## An Overview Of Exosomes

### Definition and Biogenesis of Exosomes

**Figure 1:**
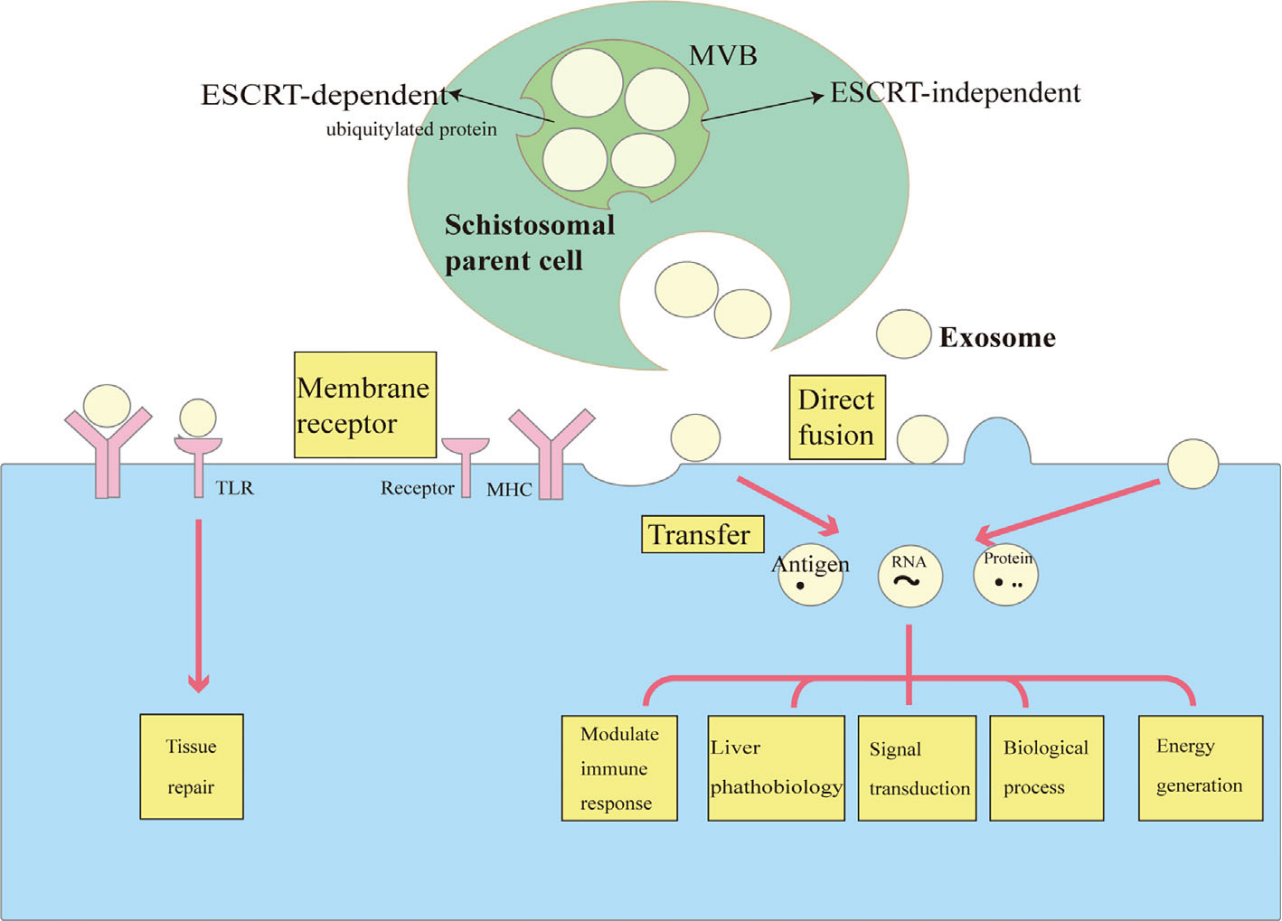
The biology and functions of exosomes in schistosomiasis. Exosomes are vesicles that are formed by the inward budding of the multivesicular body (MVB) membrane. Cargo sorting systems include an ESCRT -dependent pathway (especially for ubiquitylated protein) and an ESCRT-independent pathway (e.g., lipid raft-mediated and ceramide-dependent pathways). Exosomes are secreted following MVB fusion with the cell membrane. Exosomes interact with and are taken up by target cells in different ways, including receptor-mediated endocytosis and direct fusion with the plasma membrane. Exosomes can be regarded as delivering vehicles for several biological processes in schistosomiasis. They transfer antigens, proteins and RNA to modulate immune responses, gene expression, and metabolic processes. Exosomes are also involved in the transfer of lipids to recognize TLRs, thus participating in tissue repair.

Exosomes are extracellular vesicles generated by most cells that facilitate intercellular communication (10, 19). They were first discovered in 1983 as extracellular vesicles released by sheep reticulocytes through the exocytosis of multivesicular endosomes (20). As initially described, those exosomes were released from intraluminal vesicles (ILVs) and through a fusion of inward membrane protrusions within intracellular endosomal structures termed multivesicular bodies (MVBs) (10, 21). The ability of exosomes to interact with other cells depends on their cargoes; in understanding how the cargoes are loaded, the most extensively studied pathway is the endosomal sorting complex required for transport (ESCRT) machinery, which mainly recognizes ubiquitylated proteins. The ESCRT is composed of multi-subunit protein complexes, and by cooperatively working together, it packages membrane proteins into ILVs and generates MVBs that bind with the plasma membrane, and releases ILVs into the extracellular environment (10). Exosomes are released and ready for uptake and integration into other cells with different manners including receptor-mediated endocytosis, phagocytosis, micropinocytosis, or direct fusion with the plasma membrane. Alternatively, ESCRT-independent mechanisms such as the lipid raft-mediated and ceramide-dependent pathways may also serve as cargo sorting mechanisms (10, 22) (**Figure 1**).

### Exosome Functional Heterogeneity

Exosomes represent heterogeneous populations that share homologous features. They can all express common surface markers such as tetraspanins (CD9, CD63, and CD81), heat shock proteins (HSP 60, HSP 70, and HSP 90), biogenesis-related proteins (Alix and TSG 101), and carry messenger RNA (mRNA), microRNAs (miRNA), and long non-coding RNAs (lncRNA), and DNA, and lipids (cholesterol and ceramide) (23, 24).

One aspect of exosome functional heterogeneity is related to their parental cells as sharing similar contents during detachment; however, they differ from their original cells and constantly alter the proportion of some contents under certain pathophysiological conditions (18). For example, platelet-derived exosomes express platelet surface receptors such as CD41 (platelet glycoprotein IIb, GPIIb) and CD42b (GP1b-alpha) that can mediate platelet adhesive interactions with endothelial cells and monocytes. Remarkably, platelet-derived exosomes are able to alter their cargo contents during inflammation, for example, they increase levels of the a-granule chemokines CXCL4 and CXCL7 (25–27). This may be the mechanism that enables exosomes to induce blood cells clumping in responses to malaria (28, 29). Ongoing technological and experimental advances are likely to yield more discoveries regarding exosome functional heterogeneity.

## The Roles of Exosomes In Host–Parasite Interactions

Exosome roles in parasite diseases have been increasingly noted in recent years, including in the infection of protozoa (*Leishmania* and *Trichomonas*), fungi (*Cryptococcus neoformans* and *Paracoccidioides brasilensis*), and helminths such as *Heligmosomoides polygyrus*, *S. mansoni*, and *S. japonicum* (18, 30–34).

One of the most interesting aspects of studying exosomes in parasite infections is that both the pathogen and host make and release exosomes into the extracellular environment, and both likely play a role in disease pathogenesis. Thanks to recent scientific advances that allow effective isolation of extracellular vesicles, it is now possible to identify the contents in exosomes from parasites and host separately, which used to be a challenging aspect of their exploration (35). An endorsed model is as follows: first, parasite-derived exosomes fuse with host cells and deliver their content (e.g., proteins, miRNAs, lncRNAs, and nucleic acids) and thereby initiate host–parasite communication. Next, the host releases its exosomes to promote the activation of other immune cells such as natural killer (NK) cells, macrophages, monocytes, and lymphocytes (36). Overall, exosomes from parasites may facilitate disease pathogenesis by transferring genetic material to targeted cells; in contrast, host-derived exosomes may play a key role in host defense (18). With increasing investigations into exosomes in the field of schistosomiasis, here we review the role of exosomes in crosstalk between the host and schistosome. The underlying mechanisms could be used in parasite-control programs.

## Exosomal Cargoes In Schistosomiasis Pathophysiology

### Parasite-Derived Exosomes in Schistosomiasis

Through advanced isolation, proteomic characterization, and high-throughput sequencing techniques, researchers have successfully identified a large population of exosomal proteins and miRNAs released by *S. japonicum*, *S. mansoni*, and *S. haematobium* (33, 34, 37–40). However, the cargo profiles can vary in exosomes from the different stages, for instance, their eggs, schistosomula (<15 days), and adult worms (22–35 days) (33, 34, 37–40). Thus, understanding the components in different stages may contribute to better schistosomiasis control strategies.

#### Protein Contents Within Schistosome-Derived Exosomes

Certain proteins are shared among exosomes from different schistosomal species and different helminthes (33, 37, 38, 41). For example, many exosomal markers, such as CD63, HSP70 and 90, actin, glyceraldehyde 3-phosphate dehydrogenase (GAPDH), and Rab GTPase family members are shared by *S. japonicum*- and *S. mansoni*-derived exosomes, indicating conserved roles across different schistosome subtypes (33, 37, 38, 41, 42). Since parasites lack a classic endocrine system, exosomes could meet schistosome developmental requirements at different stages. It has been proposed that exosomes could transfer GAPDH—one of the most abundant enzymes in glycolysis—to break down glucose for energy (38). Regarding metabolic processes and the iron transport and storage proteins, ferritin isoforms are highly represented in *S. mansoni-* and *S. japonicum*-derived exosomes (37, 41), together with saposin-like proteins, which are the most abundant protein in *S. mansoni* exosomes and are used by parasites to break down red blood cell membranes and release hemoglobin, suggesting a role for exosomes in the nutrient acquisition process (41, 43, 44). More studies are underway to explore shared exosome compositions and understand their activities linked to schistosomiasis pathophysiology.

#### Small RNAs Within Schistosome-Derived Exosomes

Small RNAs account for a large proportion of exosomal cargoes and include small nuclear RNAs (snRNAs), tRNAs, miRNAs, and piwi-interacting RNAs (piRNAs), which are ~18 to 30 nt RNAs involved in transcriptional and post-transcriptional silencing of transposons (33, 45). Among these species, miRNAs are the most studied and their regulatory roles in host–pathogen interactions are increasingly clear. Several common miRNAs such as miR-10, bantam, and miR-125 have frequently been found released from exosomes of *S. japonicum* and *S. mansoni* adult worms, as well as Schistosoma eggs (37–39). By studying exosomes from different *Schistosoma* stages, researchers found that many exosomal miRNAs appear to be dominant in one stage but not in the following stage (33, 37, 39). For example, several miRNAs (miR-71, miR-7b, miR-1, miR-124, miR-0001 and miR-7) are abundant in *S. japonicum* egg-derived exosomes, suggesting that they may be involved in embryo development to cooperate with their undergoing morphological or pathophysiological changes (38, 39, 46, 47). Using a murine model, researchers confirmed that highly conserved miR-71 and Bantam were packaged in *S. japonicum* egg-derived exosomes and taken up by murine hepatocytes (39). Remarkably, miR-71 and bantam are also packaged in *H. polygyus*, *B. malayi*- and *S. mansoni*-derived exosomes, indicating that there is a conserved cargo loading system that targets specific host cells (33, 48, 49).

Adult worms and eggs can release exosomes containing parasite-specific miRNAs and conserved miRNAs that share identical sites with host genes (39). Intercellular delivery of exosome-associated small RNAs to host cells may increase parasite virulence in a fashion similar to that of the mammalian miRNA transport mechanism (39). Alternatively, exosomal miRNA transfer may target host mRNAs and therefore serve as an important mechanism for inducing epigenetic modifications in intracellular signaling and post-transcriptional regulation of host gene expression (50). To understand the biological relevance of these exosomal miRNAs secreted by schistosomes, computational analyses have been used to detect conserved regions in many of the schistosomal miRNAs, supporting the hypothesis that these could recognize human transcripts. For example, miR-125b, an abundant miRNA in *S. mansoni* exosomes, has more than 600 potential human targets based on a conserved 8-mer seed region of the mature miRNA (38). Similarly, bioinformatics analyses of murine liver cells revealed that the *S. japonicum*-derived Bantam targeted at least three host genes (Gins4, Tysnd1, and Utp3) (37). The extent to which exosomal miRNA transfer impacts recipient cells’ biological functions remains unclear. Nevertheless, the intrinsic properties of exosomes such as the ability to carry miRNA or siRNAs indicate their potential utility in the therapeutic control of many diseases (10, 51).

Another novel class of sncRNAs identified in schistosomula exosomes is the tRNA small-derived RNAs (tsRNAs) (33), which are usually 18–40 nucleotides in length and generated from precursor or mature tRNAs (45). Some of the most abundant schistosomula exosomal tsRNAs including tsRNA-Gly, tsRNA-Gln, tsRNA-Glu, tsRNA-Asp and tsRNA-Leu were previously found in exosomal cargo derived from Leishmania exosomes (52). Numerous tsRNAs may be taken up by host cells and subsequently contribute to sabotaging host defensive barriers. However, there is limited data to support this hypothesis. In particular, it remains unclear whether exosomal tsRNAs derived from schistosomula play roles in translational inhibition and/or transcriptional repression, and scientists have postulated that tsRNA may function like miRNA to participate in RNA silencing (45, 53).

#### Schistosoma-Specific Antigen Within Exosomes

Schistosoma and its released eggs can produce bioactive antigens that play critical roles in inducing host immune responses. Recent studies have implicated the participation of exosomes in delivering schistosome-specific proteins or peptides that are subsequently internalized by host DCs, macrophages, and T cells (37, 41, 54–57). In this regard, major egg antigens (p40, CAX78232) and schistosomal surface antigens (22.6-kDa tegumental antigen (AAC67308) and tegument antigen [(H)A) (CAX71406)} have been identified in *S. japonicum* exosomes (37). Determining whether they are potential vaccine antigens could help achieve schistosomiasis control. Efforts have also been made to understand how these schistosome-specific proteins are packaged within exosomes and further delivered and taken up by host cells (58). This work could provide new insights into the strategies of competently targeting exosomes applied for the antigen-presenting process. A previous study stated that certain exosomal proteins purified from the exosome-like vesicles in *S. japonicum* were able to present soluble worm antigenic preparations and activate macrophages *in vitro* (54). However, the key players responsible for macrophage activation remain obscure, and the detailed cargoes and precise mechanisms exerted by schistosome-derived exosomes require further investigation.

### Host-Derived Exosomes in Schistosomiasis

In schistosomiasis, both *S. japonicum* and *S. mansoni* eggs and adult worms contribute to host-derived exosome releases. Recent literature has confirmed host exosomal cargoes could change following schistosome infection (59, 60). Circulating exosomes in plasma are collected and isolated by centrifugation and size-exclusion chromatography, so schistosome-derived exosomes cannot be totally excluded (59). Identified by shotgun liquid chromatography/mass spectroscopy (LC-MS/MS), the protein profile in exosomes from infected hosts is different than that observed uninfected hosts, with 14 proteins exclusively detected in exosomes from *S. mansoni*-infected hosts (59). These differentiated proteins could be involved in hemostasis (fibronectin, fibrinogen gamma chain, alpha-2 antiplasmin, thrombospondin-1), extracellular matrix formation (collagen alpha-1, 2 and 3), and cytoskeleton construction (spectrin alpha chain, ankyrin-1), leading to fibrosis or other inflammatory responses (59). Exosomal lipid contents also markedly differ between infected and uninfected hosts. In exosomes from infected hosts, some major phospholipid classes including phosphatidylcholine, phosphatidylserine, and sphingomyelin, are all relatively increased along with the decrease of phosphatidylinositol (PI) and lyso-phospholipid species, reflecting a shift in exosomal origin (59). Additional *in vitro* and *in vivo* analyses are necessary to compare the detailed compositions in exosomes from different cell types.

## Schistosome-Derived Exosomes in Signal Transduction

To understand schistosomiasis pathophysiology, proteomic data of exosomes derived from *S. japonicum* and *S. mansoni* were further subjected to gene ontology-enrichment analyses. The results were strikingly consistent among different studies, showing prominent molecular functions in binding and catalytic activity, translation regulatory activity, metabolic regulation and structural molecular activity (37, 38). Additional Kyoto Encyclopedia of Genes and Genomes analyses identified many signal transduction processes in which those parasite-derived proteins participated. These pathways broadly include small GTPase-mediated signal transduction and protein transport, the ubiquitin-proteasome pathway, and inflammation-associated chemokine or cytokine signaling (37, 38). Ovchinnikov et al. analyzed the miRNAs from exosomes of *S. mansoni* adult female and male worms and predicted miRNA targets in human and cattle mRNAs (61). They found that in the hosts the Wnt pathway stood out as a primary target of *S. mansoni* RNAs; this evolutionarily conserved pathway participates in many biological processes including cell proliferation and liver physiology (62). Several target genes from hosts involved in the immune system have also been identified, suppression of which could reduce inflammatory responses (61). Studies are ongoing to determine the precise signal transduction pathways activated in response to parasitic immune invasion.

## Exosome-Mediated Host–Parasite Interactions in Schistosomiasis

This section introduces the impacts of exosomes in schistosomiasis from several aspects including cell-to-cell communication through exosomes and their multiple effects on schistosomiasis pathophysiology (illustrated in **Figure 1** and summarized in **Table 1**).

**Table 1:** Functions of the substances of host- and parasite-derived exosomes in schistosomiasis.

Source	Cargo	Target cells	Function	Reference
Parasite: schistosome	Metabolic enzymes	Host cells	Metabolic processes	(38)
	Signal proteins		Signal transduction	(38)
	Schistosomal antigens		Potential therapeutic targets	(63)
	Transport and storage proteins		Obtain nutrients	(41, 43)
	Exosomal markers		Potential therapeutic strategy	(37, 41)
	Proteasome		Potential therapeutic targets	(37)
			Biological process	
	N-glycans	moDCs	DC maturation and cytokines release	(58)
	Bantam miRNAs	Macrophages	Promote inflammatory responses	(64)
		Host cells	Potential therapeutic strategy	(37)
		Macrophages	Promote inflammatory responses	(64)
	miR-10	T helper cells	Downregulate Th2 immune response	(55)
	miR-148a	Macrophages	Reduce macrophage polarization	(65)
	sja-miR-3096	Hepatoma cells	Inhibits proliferation and migration of hepatoma cells	(66)
	sja-miR-2162	HSC	Activation and fibrogenesis	(67)
	sja-miR-1	HSC	Activation and fibrogenesis	(68)
	sja-miR-71a	HSC	Inhibit Activation and fibrogenesis	(69)
	LPC, PGD2	Eosinophils	Promote hepatic tissue repair	(70)
Host hUCMSC	Not studied	HSC	Reduce HSC activation and liver injury	(71)
Host DCs	Not studied	Not studied	Reduce inflammation responses	(72)

DC, dendritic cells; HSC, hepatic stellate cell; hUCMSC, human umbilical cord mesenchymal stem cells; LPC, lysophosphatidylcholine; miR/miRNA, micro ribonucleic acid; PGD2, prostaglandin-D2; sja, Schistosoma japonicum.

### Exosomes Play an Important Role in Macrophage Polarization in Schistosomiasis

Macrophage polarization occurs in response to pathogen infections. Polarized macrophages are usually divided into two categories: 1) classically activated macrophages (M1 subtype) characterized by high levels of CD16/32, tumor necrosis factor (TNF)-α, interleukin (IL)-12, cytokine-inducible nitric oxide synthase (iNOS) and 2) alternatively activated macrophages (M2 subtype) expressing high levels of CD206, transforming growth factor (TGF)-β1, arginase-1 (Arg-1) and IL-10 (73). Previous studies suggest these two subtypes have the opposite effect on schistosomiasis. For example, the M1 subtype plays a role in killing schistosomula through NO production and preventing hepatic fibrosis, whereas the M2 subtype contributes to schistosome-induced hepatic fibrosis though Arg-1 metabolism of L-arginine to proline and polyamine (54). Researchers used high-speed centrifugation to purify *S. japonicum* exosome-like vesicles containing special excretory/secretory proteins. They found that these structures can induce primary macrophages (RAW264.7 cells) to release iNOS and TNF-α, express CD16/32, and enhance M1 type immune-activity (54). In contrast, studies using bone marrow-derived mouse (C57BL/6) macrophages cultured with *S. mansoni* exosomes found no change in TNF-α or IL-12 production (38). Another group reported that *S. japonicum* soluble egg antigen (SEA) stimulation-induced bone marrow-derived DCs to release exosomes with subsequent increased production of TGF-β and decreased levels of TNF-α and IL-12 that attenuated the inflammation in a mouse model of acute colitis (72). It seems that host immune cells could initiate their own anti-inflammatory mechanism by producing exosomes, whereas parasite-derived exosomes may target specific host cells displaying pro- or anti-inflammatory effects. Furthermore, the *in vitro* studies suggest that different species of schistosomes or protocols used for exosome preparations may contribute to their functional discrepancies (38).

As mentioned above, miRNAs constitute one of the most important exosomal cargoes to regulate host–parasite interactions (74). A recent report verified that macrophages ingest *S. japonicum*-derived exosomes containing high contents of miR-148a, which may target phosphoinositide 3-kinase/protein kinase B pathway leading to upregulation of TNF-α, IL-12, Arg-1, and IL-10 (65). Using RAW264.7 cells, Liu et al. found *S. japonicum* exosomes containing parasite-specific miRNAs (miR-125b and Bantam) are functionally relevant in the recipient RAW264.7 cells because they up-regulate the number of monocytes and TNF-α expression (64). They further identified protein S1 in RAW247.1 cell as the downstream target of sj-miR-125b, which promoted the activation of Toll-like receptor (TLR) signaling during monocyte proliferation. Moreover, *Clmp* and *Fam212b*, are considered downstream mRNAs of sj-Bantam to enhance TNF-α production. As result, transfecting RAW264.7 cells with helminth-specific miRNA cargoes promoted parasite survival and increased worm burden and egg deposition in mice models. Given that miR-125b was confirmed to positively regulate the M1-phenotype (75), further studies are still required to clarify whether *S. japonicum*-derived exosomes containing miR-125b and Bantam can indeed promote M1 polarization.

### Exosomes Regulate the Th2 Immune Response in Schistosomiasis

Schistosoma adult worms constantly lay eggs in the host venous systems, and both stages modulate the Th2 immune response in chronic schistosomiasis (1). Exosomes may attenuate T cell differentiation. Researchers isolated T helper cells from Peyer’s patches and mesenteric lymph nodes of *S. mansoni*-infected mice and found that they contain schistosomal miRNAs including miR-10, bantam and miR-15 that were packed in the *S. mansoni* adult-derived exosomes and taken up by Th cells (55, 76). The exosomal miR-10 can suppress the downstream molecule mitogen-activated protein kinase kinase kinase 7 (MAP3K7, also known as TAK1) and subsequently affect the nuclear factor-kB pathway in Th cells, consistent with the effect of live schistosomes on Th cells (55). When co-cultured with schistosomes, differentiation toward Th2 pathway was reduced, and the expression levels of associated genes such as IL-33, Tnfsf4, Tim-2, and Anxas were decreased, while the polarizing factors toward the Th1 pathway (e.g., Tim-3) were increased (55, 76). Besides Th cells, DCs also contribute to immune responses (77). Glycosylated exosomes from *S. mansoni* schistosomula can be internalized by human monocyte-derived DCs (moDCs) though recognizing the DC-specific ICAM-3-grabbing nonintegrin (DC-SIGN, CD209) on the DC membrane (58). This uptake of exosomes can augment moDC maturation to increase IL-10 release and programmed death-ligand (PD-L)1 expression (58). PD-L1 is involved in inhibiting CD4^+^T cell activation and participating T cell tolerance, and the immunity tolerance may help to explain how schistosomula or cercariae escape host protective immune responses and develop into mature forms (78–81). Considering the diversity of immune cells such as NK cells and neutrophils, it needs to be further proved whether the same Th2 polarization inhibition and/or relevant cytokine release occur in other types of immune cells following co-culture with schistosome-derived exosomes.

### Exosomes Are Engaged in the Process of Egg-Induced Granuloma to Liver Fibrosis of Schistosomiasis

**Figure 2:**
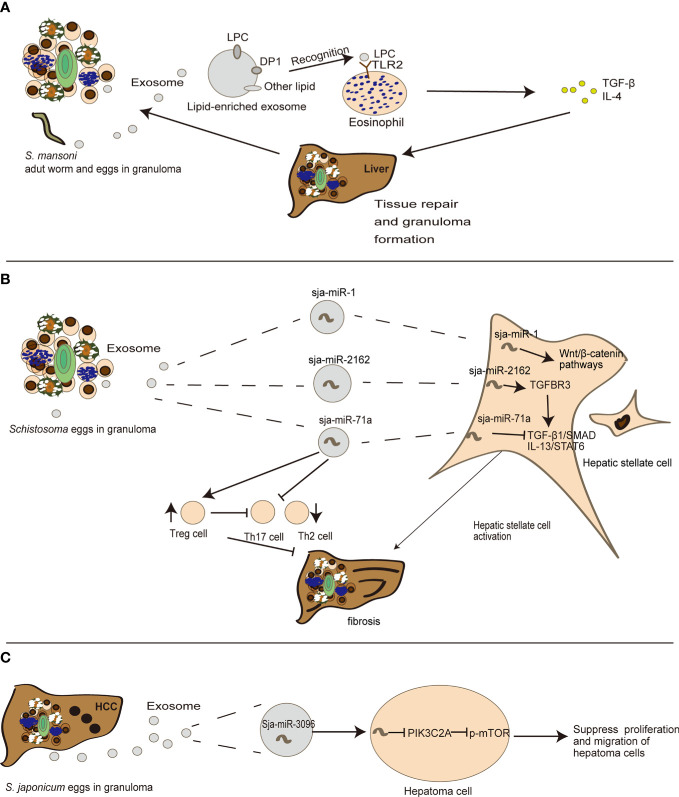
The detailed mechanism of the role exosomes play in the evolution of egg-induced granuloma to liver fibrosis. **(A)** *S. mansoni* egg-derived exosomes can promote eosinophil activation, transferring LPC (lysophosphatidylcholine) to recognize TLRs (Toll-like receptors) on eosinophils, thus participating in tissue repair. **(B)** *S. japonica* egg-derived exosomes can carry sja-miR-71a, sja-miR-1 and sja-miR-2162 to modulate HSC activation, thereby participating in the process of liver fibrosis. **(C)** sja-miR-3096 in *S. japonicum* egg-derived exosomes can suppress hepatoma proliferation and migration.

Tissue repair and inflammation can promote liver fibrosis in several diseases including alcoholic liver disease and chronic hepatitis (4). Emerging evidence also indicates that exosomes can participate in liver fibrosis (21). In schistosomiasis, Coakley et al. found that *S. mansoni* egg-derived exosomes can promote the activation of eosinophils, an important event in granuloma formation (70). Exosomes carry lysophosphatidylcholine and prostaglandin (PG) D2 derived from schistosome tegument, to target TGF-β and bind lipid receptors on eosinophils, including TLR2 and DP1 (a PGD2 receptor), thereby triggering subsequent liver tissue repair (70) (**Figure 2A**). This may in turn cause chronic inflammation of schistosomiasis, and construct a fibrogenic feedback loop (4). Besides eosinophils, hepatic stellate cells (HSCs) also serve as critical modulators of hepatic fibrosis pathophysiology and schistosomiasis development (82). He et al. revealed that sja-miR-2162 present in *S. japonica* egg-derived exosomes are abundantly detected in HSCs and stimulate the activation of HSCs by upregulating the expression of fibrosis-related proteins, such as collagen 1α1 (*Col1α1*), collagen 3α1 (*Col3α1*), and α-smooth muscle actin (*α-Sma*) (67). Sja-miR-2162 can directly target the TGF-β receptor III involved in TGF-β signaling in HSCs (67). Another experiment revealed that *S. japonica* egg-derived exosomes are internalized by HSCs, and the major cargo sja-miR-1 in *S. japonica* egg-derived exosomes can activate the Wnt/β-catenin signaling to promote hepatic fibrosis (67, 68). Notably, sja-miR-2162 and sja-miR-1 are not the most abundant substances in schistosome-derived exosomes, but they can be detected in host HSCs (67, 68), implying a selective exosomal miRNA sorting mechanism into host cells. Interestingly, a recent study found that *S. japonicum* egg-derived exosomes containing a higher abundance of sja-miR-71a could directly inhibit HSC activation *in vitro*, thereby attenuating liver fibrosis by targeting the TGF-β1 and IL13 axis (69). This finding is contrary to previous reports about free sja-miRNA (67, 68). Meanwhile, *S. japonicum* egg-derived exosomes could downregulate Th2 and Th17 cells and increase Treg cells in *S. japonicum*-infected mice (69). It is notable that Th2 and Th17 cells can promote liver fibrosis (5), but Treg cells can inhibit T cell function (69, 83) (**Figure 2B**). These results suggest that instead of directly interacting with HSCs, schistosome-egg or SEA may stimulate other cells including hepatocytes or Kupffer cells in the liver to modulate fibrosis progression. Exosomes may also play an important role in host–cell interactions and antigen presentation in the infected liver in schistosomiasis, although there is limited research in this field.

Liver fibrosis is a chronic process that can lead to hepatocellular carcinoma (HCC). Emerging evidence shows that exosomes play a role in this stage (84). There is not sufficient evidence for a direct relationship between schistosomiasis and HCC. Notably, a recent study indicated that *S. japonica*-derived exosomes containing sja-miR-3096 can be internalized by hepatocytes and subsequently suppress hepatoma cell proliferation and migration, although there was a limited effect on the cell cycle of normal liver cells, supporting its controversial role in carcinogenesis (66) (**Figure 2C**). Interestingly *S. mansoni* antigens from trapped eggs in granulomas can initiate the activation of two critical regulators related to hepatocellular carcinogenesis in adjacent human hepatocytes: c-Jun and signal transducer and activator of transcription 3. These genes may further induce DNA- double-strand breaks and subsequent oncogene activations as a mechanism shared by other pathogens (85). The progression of egg-induced granuloma to liver fibrosis and even HCC is illustrated in **Figure 2**.

## Application of Exosomes in Schistosomiasis Diagnosis

Exosomes are promising biomarkers for the diagnosis and prognosis of several diseases and may contribute to the development of minimally invasive diagnostics and next-generation therapies (86, 87). However, it is important to accurately define homogeneous exosome populations before embarking on large-scale production for detailed biochemical analyses and preparation of clinical-grade reagents. Here, we review the methods for exosome isolation and characterization and possible applications related to schistosomiasis.

### Exosome Isolation and Detection Methods

Five groups of exosome isolation techniques have been developed: 1) differential ultracentrifugation-based techniques, which are the most commonly used method; 2) size-based techniques, such as ultrafiltration; 3) immunoaffinity capture-based techniques; 4) exosome precipitation techniques; and 5) microfluidics-based techniques (12). After isolation, common methods to characterize their physical features include: 1) ultrastructural analysis, with microscopy-based methods, such as scanning electron microscopy (SEM), transmission electron microscopy (TEM), and atomic form microscopy (AFM) (88); 2) nanoparticle tracking analysis (NTA) using a light beam to determine concentration and size distribution of particles to identify the exosomes; 3) asymmetric flow field-flow fractionation (AF4), which identifies nanoparticles based on their density and hydrodynamic properties through two perpendicular flows (89); and 4) flow cytometry, which requires micrometer-sized latex bead to bind to small exosome particles that are subsequently stained with fluorescent antibodies and characterized for their protein markers (88).

Initially, techniques such as ultracentrifugation and ultrafiltration were used to separate and purify exosomes from samples (90). However, in practice, it is difficult to analyze only exosomes with these techniques because the sample also contains other items such as particles with similar characteristics and concentrations and high-weight proteins (91). Hence, this method is sometimes combined with affinity purification (91). In the final stage, protein analyses including western blotting, enzyme-linked immunosorbent assays (ELISA) and mass spectrometry are frequently applied to determine the physio-pathological functions (91). In recent years, a new generation of biosensors was introduced to detect a wide range of exosomes with different sizes and molecular contents. The newly developed platforms include bead-based flow cytometry, small particle flow cytometry, ExoScreen (based on photosensitizer beads technology), and iMEX (based on an integrated magnetic-electrochemical sensor) (91).

### Substances in Exosomes Have Diagnostic Value for Schistosomiasis

Exosomes could be used for the definitive diagnosis of schistosomiasis. Several studies have investigated the diagnostic roles of proteins and peptides (92, 93). Using extensive vortexing, centrifugation, and ultracentrifugation steps to isolate urinary exosomes from patients, researchers detected lower expression of aquaporin-2 and higher Na-K-2Cl cotransporter expression in exosomes from hepatosplenic schistosomiasis (HSS) patients. This suggested that urinary exosomes may be used to diagnose renal tubular abnormalities of HSS patients (60). Chen et al. concluded that detecting combined epitope proteins from exosomes including saposin B domain-containing protein and BAR domain-containing protein, has a modest sensitivity to detect schistosomiasis (94). In addition to proteins, miRNAs are considered an ideal marker of schistosomiasis. Meningher et al. found that the sensitivity and specificity of detecting parasite miRNAs including Bantam, miR-2c-3p, and miR-3488 in exosomes are much higher than other diagnostic methods, with the combination of Bantam and miR-2c-3p yielded a sensitivity and specificity of 91 and 94.12%, respectively (95). This indicates that the combination of different molecules such as parasite-specific miRNAs in exosomes could improve accuracy compared to RNA alone. There are also some patents registered for serum exosomal miR-142-3p and miR-223-3p as diagnostic markers of schistosomiasis (Patent number: CN110760589-A and CN110760590-A) (96, 97). Although the exact roles of these miRNAs in schistosomiasis are unknown, they have been associated with other diseases (98, 99). Future research could clarify the origins and amounts of exosomal miR-142-3p and miR-223-3p to verify their diagnostic value.

Some studies also reported that exosomal products can serve as prognostic and stage-predictive biomarkers of schistosomiasis (95, 100). From cercariae penetrating the skin to adult worms residing in portal systems and laying eggs, the substances in exosomes from different stages of schistosomes have variations and change with disease progression (33, 37–39, 41). Cai et al. found a negative correlation between the serum level of exosomal miR-146a-5p and liver fibrosis grades in patients with schistosomiasis. The level of serum exosomal miR-146-5p in healthy subjects or patients with mild hepatic fibrosis (grades 0–I) caused by schistosomiasis is higher than in patients with moderate to severe (grades II–III) liver fibrosis, indicating that miR-146a-5p could be applied as a novel discrimination marker for schistosomiasis severity (100). Furthermore, compared with serological tests, Bantam and miR-2c-3p become negative within 32 weeks after treatment, which strongly suggests their potential applications for follow-up (95). The potential diagnostic components of exosomes in schistosomiasis are summarized in **Table 2**.

**Table 2:** Exosomal substances from the serum of infected patients can be used as diagnostic tools in schistosomiasis.

Origin	Substances	Function	Reference
Schistosome	Combined epitope protein	Definitive diagnosis	(94)
	Bantam miRNAs		(95)
	miR-2c-3p		
	miR-3488		
Host	miR-142-3p		(96)
	miR-223-3p		(97)
	miR-146a-5p	Hepatic fibrosis grade	(100)
	AQP2, NKCC2	Diagnosis for renal tubular abnormalities	(60)

AQP2, aquaporin-2.

Because exosome numbers are limited in the serum of patients and exosome extraction and purification technologies are not very mature, it remains difficult to accurately identify exosomes and effectively detect the substances they contain. Schistosome-derived substances in host cells are promising targets but remain a long way off in clinical practice.

## Application Of Exosomes in Schistosomiasis Therapy

Although praziquantel effectively kills adult schistosomes, it does not kill immature forms and has little effect on schistosomiasis once the liver sustains a chronic or severe injury (101, 102). Alternative approaches to treat this neglected disease are needed. Exosomes are promising biomarkers for the diagnosis and prognosis of various diseases and may contribute to the development of minimally invasive diagnostics and next-generation therapies for schistosomiasis. However, it is important to accurately define a homogeneous exosome population before embarking on large-scale production for detailed biochemical analysis and preparation of clinical-grade reagents preparation (10). This section summarizes the potential applications of targeting exosomes to interrupt schistosomiasis, which may reduce disease severity.

### The Specific Cargoes in Schistosome-Derived Exosomes Can Be Applied as Potential Vaccine Candidates

It is well-known that lipid metabolism inhibitors like GW4869 can efficiently prevent exosome release in a variety of cells *in vitro* (103, 104). However, clinicians should be cautious with the direct usage of these drugs *in vivo* since they have cytotoxic side effects (105). With the growing evidence that exosomes carry diverse representative biomolecules and affect intercellular communication, schistosome-derived exosomes are being studied to identify vaccine candidates and improved treatments.

In 2016, Zhu and colleagues conducted the first proteomic characterization study of *S*. *japonicum* extracellular vesicles (37). Isolated exosomes were subjected to morphological analysis using transmission electron microscopy, and the protein components were determined by LC-MS/MS analysis, resulting in the identification of 403 proteins (37). Subsequent bioinformatics analyses determined that proteins including the ubiquitin–proteasome system could play a major role in schistosome invasion, further indicating that these proteins could be candidate targets for anti-schistosome therapies. In 2018, Samoi et al. conducted the first report of exosomal protein and miRNA secretion from adult *S. mansoni* (38); 130 schistosome proteins were identified including previously described vaccine candidates, such as glutathione-S-transferase (GST), tetraspanin (TSP-2), and calpain. Researchers have verified that anti-Sm-TSP-2 antibodies could block *S. mansoni* -secreted exosomes uptake by both human endothelial cells and monocytes, which could induce differential expression of genes mainly involved in arachidonic acid metabolism, inflammation and blood clotting (106). It was promising to observe when *S. haematobium*-infected mice were vaccinated with TSPs, there were significantly reduced egg burdens in the liver and intestine (44). More studies on transcriptional profiles related to parasite-derived exosomes were recently published. For example, 143 miRNAs associated with *S. mansoni* exosomes were identified through matching to platyhelminth (flatworm) miRNAs in the miRbase database (38). A recent biogenesis study of *S. japonicum* suggests that exosome gene profiles vary with a developmental stage (40), indicating that targeting stage-specific proteins or RNAs in exosomes as vaccine candidates could be beneficial to both the early prevention and treatment of schistosomiasis in different periods. The cargo profiles from schistosomes in different developmental stages were reviewed in previous sections (33, 34, 37–40). In addition, one study proposed that fatty acids and sterols could be targeted for anti-schistosome interventions because parasites cannot *de novo* synthesize these molecules (107). The vaccine candidates associated with schistosomal exosomes are summarized in **Table 3**. These exosome-derived vaccine candidates could ameliorate the pathology and blocking schistosomiasis transmission.

**Table 3:** Exosomal substances as potential vaccine targets.

Type of vaccine	Identified name	Exosomes from *S. mansoni* adult worm (41)	Exosomes from *S. mansoni* schistosomula (33)	Exosomes from *S. japonicum* adult worm (37)	*S. mansoni* egg (108)
Identified vaccine target (41)	Enolase	√	√	√	√
	Glyceraldehyde-3-phosphate dehydrogenase	√	√	√	√
	Glutathione S-transferase 26 kDa	√	√	√	√
	Calpain	√	√	√	√
	Leucine ami peptidase	√			
	14-3-3 protein	√	√		√
	Annexin	√	√	√	√
	Tetraspanin	√	√	√	
	Cytoplasmic dynein light chain	√			√
	Dynein light chain	√	√	√	√
	Syntenin	√			
	Antigen Sm21.7	√	√		√
	Tegument antigen 22.6	√		√	
	Sm29	√	√		
	Heat shock protein 70	√	√	√	√
	Saposin B domain-containing protein	√		√	√
	Thioredoxin peroxidase	√			
	Ectonucleotide pyrophosphatase/phosphodiesterase	√			
Potential vaccine target (107, 109, 110)	Lipid	√	√	√	√
	Sterol	√	√	√	√
	Triose Phosphate Isomerase	√	√	√	√
	Glyceraldehyde-6-Phosphate Dehydrogenase	√			
	programmed cell death protein		√		
	Adenosylhomocysteinase, putative	√		√	√
	Glutathione S-transferase 28 kDa	√	√	√	√
	Glucose transport protein	√			
	Tubulin	√		√	√
	Histone H3	√			√
	Histone H4	√		√	√
	Histone H2B	√			√
	Chaperonin containing t-complex protein 1	√			
	T-complex protein 1 subunit alpha			√	√

Common schistosomal vaccine targets detected in different exosome species. Vaccine targets are classified according to the specific stages and types of exosome origins. If the target exists in relevant exosomes, it is a tick; if not, it is blank.

### Host-Derived Exosomes in Schistosomiasis Therapies

Many groups are studying host-derived exosomes in pathophysiological conditions due to their potential clinical applications. A recent study found that DC-derived exosomes modulate immune responses and prevent the development of autoimmune diseases (72). In a mice inflammatory bowel disease model, Wang et al. found that DC-derived exosomes treated with SEA attenuated dextran sulfate sodium induced colitis in mice (72). The authors proposed that DC-derived exosomes may contain a range of nanometer-sized membrane vesicles including the surface expression of co-stimulatory molecules, functional major histocompatibility complex-peptide complexes and other immune function-associated molecules that may modulate anti-inflammatory responses (e.g., inducing TGF-β expression) and in contrast, suppress the expression of inflammatory cytokines (e.g., TNF-α, IFN-α, IL-17A, IL-12, and IL-22) (72). Furthermore, Dong and colleagues found that exosomes released by human umbilical cord-derived mesenchymal stem cells (hUCMSCs) could be more efficient than the cells themselves for suppressing HSC activation, improving hepatic cell regeneration, and reducing liver injury and fibrosis in *S. japonicum*-infected mice (71).

## Conclusion and Prospects

Exosomes play significant roles in various biological functions, including biomolecule transfer, and the regulation of numerous physiological and pathological processes in various diseases. Here, we reviewed the exosomal cargoes among the different stages of *S. japonicum* and *S. mansoni*, and characterized the current understanding of schistosome- and host-derived exosomes in schistosomiasis. This review is the first to introduce the roles of exosomes in the process of egg-induced granuloma progression to liver fibrosis. Although several studies have investigated the relationships between parasite-derived exosomes and immune responses in schistosomiasis, research on exosomes and liver pathophysiology by exosomes is limited. This may be explained by the fact that liver fibrosis is a late stage of schistosomiasis, unlike immune modulation as an earlier response. Finally, existing evidence suggests that exosomes can be used for diagnosis and therapy in schistosomiasis. Although clinical application and efficacy still need to be discussed, they may evolve with exosome separation technology. Meanwhile, mice vaccinated with the exosome marker anti-tetraspanins have reduced egg burden and protection against *S. mansoni* infection (44). More samples are needed, and further clinical trials should be designed to verify the efficacy of exosome therapy. With more research, it is believed that the detailed pathophysiological mechanisms will help clarify how exosomes can be applied in clinical settings for the early diagnosis, treatment and prognostic monitoring of schistosomiasis.

## Author Contributions

YY designed the outline of the manuscript, wrote, and reviewed the manuscript. JZ wrote and reviewed the manuscript. MC, HL, XL, and BZ revised the manuscript. XC and QC discussed the topic and outlines of the manuscript and reviewed the text. All authors contributed to the article and approved the submitted version.

## Funding

This work was supported by the National Natural Science Foundations of China (No. 81974077 to QC).

## Conflict of Interest

The authors declare that the research was conducted in the absence of any commercial or financial relationships that could be construed as a potential conflict of interest.
